# Evaluation of United States grain sorghum in lactation diets on sow and litter performance

**DOI:** 10.1093/tas/txag164

**Published:** 2026-10-23

**Authors:** Grady A Privett, Katelyn N Gaffield, Emily G Skelton, Jason C Woodworth, Jordan T Gebhardt, Robert D Goodband, Mike D Tokach, Joel M DeRouchey, Sierra M Collier, Kyle F Coble, Larissa L Becker, Hilario M Cordoba, Morgan A Schaeperkoetter

**Affiliations:** Department of Animal Sciences and Industry, College of Agriculture, Kansas State University, Manhattan, KS 66506-0201, United States; Department of Animal Sciences and Industry, College of Agriculture, Kansas State University, Manhattan, KS 66506-0201, United States; JBS Live Pork, Greeley, CO 80634-9039, United States; Department of Animal Sciences and Industry, College of Agriculture, Kansas State University, Manhattan, KS 66506-0201, United States; Department of Diagnostic Medicine/Pathobiology, College of Veterinary Medicine, Kansas State University, Manhattan, KS 66506-0201, United States; Department of Animal Sciences and Industry, College of Agriculture, Kansas State University, Manhattan, KS 66506-0201, United States; Department of Animal Sciences and Industry, College of Agriculture, Kansas State University, Manhattan, KS 66506-0201, United States; Department of Animal Sciences and Industry, College of Agriculture, Kansas State University, Manhattan, KS 66506-0201, United States; Department of Animal Sciences and Industry, College of Agriculture, Kansas State University, Manhattan, KS 66506-0201, United States; JBS Live Pork, Greeley, CO 80634-9039, United States; JBS Live Pork, Greeley, CO 80634-9039, United States; JBS Live Pork, Greeley, CO 80634-9039, United States; JBS Live Pork, Greeley, CO 80634-9039, United States

**Keywords:** Grain sorghum, lactation, litter performance, sow

## Abstract

A total of 568 sows (Line 1050, PIC Genetics, Hendersonville, TN) and their litters (PIC 337 × 1050) were used to evaluate the effects of United States grain sorghum compared to corn in lactation diets on sow and litter performance. Sows, in four separate farrowing groups (approximately 142 ± 1 sows per group), were moved into farrowing rooms and blocked by parity on d 112 ± 1 of gestation. Sows were assigned to one of three dietary treatments, consisting of diets with corn, grain sorghum, or a 50:50 blend of both serving as the grain source. The grain sorghum used was tannin-free and sourced from the Texas and Oklahoma panhandles. Diets were formulated to be balanced for amino acids, minerals, and net energy. Corn and sorghum were given the same net energy value (2672 kcal/kg). Sows were fed a wheat-based gestation diet, then hand-fed 0.90 kg of their treatment diet twice a day, starting 3 to 5 d pre-farrow, and were given ad libitum access from farrow until weaning (approximately d 19 of lactation). Particle size of grain and complete feed were analyzed. Grain sorghum was slightly finer than corn, reducing dietary particle size as sorghum inclusion increased; however, all diets remained within a 30 μm range. Replacing corn with grain sorghum in the diet did not affect sow weight, backfat, or caliper score change from entry to weaning. Feeding sows grain sorghum did not affect sow average daily feed intake, litter feed efficiency, or wean-to-estrus interval. There were no differences in total born or born alive. However, there was a decrease (linear *P* < 0.001; quadratic, *P* = 0.003) in the percentage of stillborn pigs with increasing grain sorghum in the diet. Also, the percentage of mummies increased and then decreased (quadratic, *P* = 0.001) as grain sorghum inclusion increased compared to corn. There were no differences in litter size or litter weight after cross-fostering (d 1) or at weaning. Preweaning mortality tended to decrease then increase (quadratic, *P* = 0.080) with increasing grain sorghum compared to corn in the diet. As grain sorghum increased, preweaning removals increased (linear, *P* = 0.046); however, no differences were observed in total removals and mortality. In conclusion, grain sorghum grown and harvested in the United States can replace corn in lactation diets with little impact on sow or litter performance.

## Teaser

There were minimal effects on sow and litter performance when grain sorghum replaced corn in lactation diets.

## Introduction

Feed represents the largest component of production costs in the swine industry. Corn is the most common cereal grain (energy source) used in swine diets in the United States. Therefore, identifying alternative cereal grains, when economically viable, that can partially or completely replace corn without negative effects on pig performance is important for improving efficiency in swine systems.

Grain sorghum is commonly grown across various regions of the United States due to its drought tolerance (Schober and Bean 2008). Because of increased production in the Great Plains region, grain sorghum can have an economic advantage compared to corn in swine diets. Nutritionally, grain sorghum contains similar concentrations of energy compared with corn, making it a potential alternative cereal grain for swine diets (NRC 2012). Historically, grain sorghum has been associated with differences in feed intake due to the tannin concentration (Cousins et al. 1981). However, 99% of the grain sorghum currently grown in the United States is tannin-free. Sorghum is defined as being tannin-free when values are at or below 4.0 mg of catechin equivalents per g of sample (U.S. Grains and Bioproducts Council 2026). Additionally, advancements in feed processing and the use of standardized ileal digestible amino acids in diet formulation have improved the nutritional value and overall usability of grain sorghum in swine diets. Multiple studies have evaluated grain sorghum as a replacement for corn in nursery and finisher pigs, and have shown that grain sorghum is a suitable replacement for corn and can be added with few negative effects on pig performance (Benz et al. 2011; Thomas et al. 2020).

During lactation, sows have high nutrient requirements to support milk production and maintain body condition (Tokach et al. 2019). The choice of dietary ingredients providing energy may influence both sow and litter performance. Previous studies have evaluated grain sorghum in lactation diets and reported variable effects on sow feed intake, body weight change, and litter performance (Louis et al. 1991; Johnston et al. 1998; Kim et al. 2002; Sotak-Peper et al. 2015). However, these studies were conducted under small-scale university conditions with sows that had much smaller litters and lighter litter weaning weights compared with current production standards in addition to using different varieties of sorghum ranging from bronze to red in color. Therefore, additional research is needed to evaluate modern tannin-free cultivars of grain sorghum as an energy source in commercial lactation diets. The objective of this study was to evaluate the effects of using United States grain sorghum as a partial or complete replacement of corn in lactation diets on sow and litter performance under commercial conditions.

## Materials and methods

The Kansas State University Institutional Animal Care and Use Committee approved the protocol used in this experiment (IACUC #4915). This study was conducted at a commercial sow farm in northwest Texas (JBS Live Pork, Dalhart, Texas). Sows were housed in individual farrowing stalls measuring 2.1 × 1.5 m that included a sow and piglet area in an environmentally controlled, mechanically ventilated barn. A heat lamp was included for piglet comfort. Each stall was equipped with a SowMax feed hopper (HogSlat, Newton Grove, NC). Two feeder types were used: a dry or a wet-dry feeder (HogSlat, Newton Grove, NC). All stalls were equipped with a supplementary piglet water nipple 16.5 cm from the floor. The dry feeder included a sow water nipple located 40.5 cm from the floor, whereas the wet-dry feeder included a water nipple located 30.5 cm from the floor, with an additional water source provided within the feed pan. Dietary treatments were evenly distributed across feeder configurations, and feeder configuration did not influence the response to dietary treatment.

### Animals and treatment structure

A total of 568 sows (average parity 2.5, Line 1050, PIC, Hendersonville, TN, United States) and litters (PIC 337 × 1050) were used across four farrowing groups (approximately 142 ± 1 sows per farrowing group) housed in four separate farrowing rooms from September to November 2025. To prevent any bias towards a specific grain, sows were offered a common wheat-based gestation diet to eliminate any potential concern of an interaction between gestation grain source and lactation grain source. On d 112 ± 1 of gestation, sows were moved into farrowing rooms, blocked by parity, and assigned to dietary treatments, which were fed from entry into the farrowing house until weaning. Parity blocks included: first parity, parity 2 to 4, and parity 5 and greater. During each placement, first parity sows were assigned to the two middle rows of the room, while multiparous sows filled the remaining stall spaces. Feeder type and treatments were randomly allocated throughout the room to minimize potential location effects.

Dietary treatment consisted of diets with corn, grain sorghum, or a 50:50 blend of both serving as the grain source (Table 1). The grain sorghum used in the study was assumed to be tannin-free and sourced from the Texas and Oklahoma panhandles. There were 189 or 190 sows for each dietary treatment. From entry until farrowing, sows were hand-fed 0.90 kg twice a day, after which feed was offered ad libitum. No creep feed was offered to piglets throughout lactation. Diets were fed in mash form and formulated to be balanced for amino acids, minerals, and net energy (NE) using NRC (2012) nutrient values for ingredients, except for NE of grain sorghum. Corn and grain sorghum were given the same estimated NE value in diet formulation (2672 kcal/kg). Both grains were ground using a twin-rotor hammer mill (Model E3820TF; Bliss Industries Inc., Ponca City, OK) equipped with a 12-mm screen followed by a 10-mm screen with a consistent hammer tip speed to achieve a target particle size of 500 μm. Grain particle size was measured after each grain grinding event and all complete diets were sampled and analyzed after delivery to the farm. Particle size of grain and complete feed were analyzed with a 13-sieve system with the inclusion of a flow agent and a 15-min run time following standard sieving procedures (Table 2; Stark and Kalivoda 2016). All diets were manufactured and particle size was analyzed at the JBS Dalhart Feed Mill (Dalhart, TX).

**Table 1:** Composition of lactation diets (as-fed basis).^a^

Item	Grain source		
	Corn	50:50 blend	Grain sorghum
**Ingredient, %**			
** Sorghum**	—	34.10	68.15
** Corn**	68.02	34.03	—
** Soybean meal, 48.2 CP%**	26.17	26.09	26.08
** Soybean oil**	1.50	1.50	1.50
** Monocalcium phosphate, 21 P%**	0.63	0.59	0.55
** Limestone**	1.80	1.82	1.83
** Salt**	0.60	0.60	0.60
** Lysine, 62.4%^b^**	0.40	0.41	0.42
** DL-Met**	0.07	0.09	0.10
** L-Thr**	0.12	0.11	0.10
** L-Trp**	0.02	0.01	—
** L-Val**	0.03	0.01	—
** Sow premix^c^**	0.25	0.25	0.25
** Feed mitigant^d^**	0.27	0.27	0.27
** Phytase^e^**	0.04	0.04	0.04
** Choline chloride**	0.11	0.11	0.11
** Total**	100	100	100
**Calculated analysis**			
**SID AA, %**			
** Lys**	1.05	1.05	1.05
** Ile:Lys**	62	64	67
** Leu:Lys**	133	140	147
** Met:Lys**	30	31	32
** Met and Cys:Lys**	54	54	54
** Thr:Lys**	64	64	64
** Trp:Lys**	20	20	20
** Val:Lys**	72	72	72
** His:Lys**	41	40	39
**Net energy (NE), kcal/kg**	2496	2498	2498
**SID Lys:NE, g/Mcal**	4.21	4.20	4.20
**CP^f^, %**	17.8	18.7	19.6
**Ca, %**	0.87	0.87	0.87
**P, %**	0.48	0.48	0.48
**STTD^g^ P, %**	0.38	0.38	0.38

a Feed was manufactured by a commercial feed mill (JBS, Dalhart, TX).

b BioLys (Evonik Industries AG, Essen Germany).

c OptiPhos Plus 2500 G (Huvepharma, Peachtree City, GA) provided 1080 FTU/kg and an expected STTD P release of 0.12% as part of the sow premix. Provide per kg of diet: 11,023 IU vitamin A; 1500 IU vitamin D; 73.5 IU vitamin E; 3.3 mg vitamin K; 0.03 mg vitamin B12; 44.1 mg niacin; 27.6 mg pantothenic acid; 8.3 mg riboflavin; 0.22 mg biotin; 2.2 mg folic acid; 1.1 mg pyridoxine; 125 mg Zn from zinc sulfate; 110 mg Fe from iron sulfate; 50.0 mg Mg from manganese oxide; 25.0 mg Cu from copper sulfate; 0.35 mg I from calcium iodate; 0.30 mg selenium from sodium selenite; 0.20 mg Cr from chromium picolinate.

d Guardian liquid (Alltech Inc, Nicholasville, KY) supplied lactic acid, propionic acid, and essential oils.

e OptiPhos Plus 2500 G (Huvepharma, Peachtree City, GA) provided 1000 FTU/kg of diet for an estimated release of 0.12% STTD P in addition to the phytase includes as part of the premix.

f CP, Crude protein.

g STTD, Standardized total tract digestibility.

**Table 2:** Average particle size of grain sources and complete diets.^a^

Item	Grain		Complete diet		
	Corn	Sorghum	Corn	50:50 blend	Grain sorghum
**Particle size, μm^b^**	504	442	597	580	571
**Geometric standard deviation^c^**	3.6	3.4	3.1	3.0	2.9

a Values represent the mean of 7 samples collected throughout the study. Particle size was determined using a 13-sieve system with the inclusion of a flow agent and a 15-min run time, following standard sieving procedures (Stark and Kalivoda 2016).

b Values reported as mean particle size (*d_gw_*).

c Values reported as geometric standard deviation (*S_gw_*).

Upon entry into the farrowing house and at weaning, sow weight, caliper score, and backfat were measured. Caliper scores were taken at the last rib (Knauer and Baitinger 2015). Backfat was measured at the last rib, 10 cm from the midline on the left side of the sow (Renco Lean Meter, S.E.C. Repro Inc., Golden Valley, MN). Daily feed additions were recorded for each sow using a scale cart and the LeeO system (Merck Animal Health, Madison, NJ). Each farrowing crate had an attached radio frequency identification (RFID) tag which identified its location in the LeeO system. When sows were moved into the farrowing stall, they were registered by location in the LeeO system.

Refused feed was removed from the feed pan at weaning, weighed, and subtracted from the recorded feed disappearance for each sow. The dry matter (DM) content of the recovered feed was analyzed to calculate feed removed on a DM basis, and diet DM was subsequently used to convert feed removed to an as-fed basis for accurate adjustment. Approximately 28% of all sows had refused feed, with the percentage of sows with refused feed at weaning being similar across dietary treatment. Additionally, feeder measurements were taken at farrowing to account for feed remaining in the feeder. The feeder measurement at farrowing was added to the sow’s total intake, whereas the feeder measurement at weaning was subtracted from the sow’s total intake.

The number of total born, born alive, stillborn, and mummified piglets was recorded for each sow throughout farrowing. Cross-fostering occurred across dietary treatments to equalize litters within 24 h of farrowing. Piglets were weighed as a litter on d 1 post cross-fostering and the day before weaning. Litter feed efficiency was calculated by dividing the total litter weight gain by the total sow feed intake from farrow to weaning. Starting on d 3 of lactation, piglets that were gaunt were removed as a fall-behind pig. All pigs removed and mortalities were weighed and recorded. After weaning, the insemination date was recorded (CloudFarms system, BASF, Florham Park, NJ, United States) and used to calculate the wean-to-estrus (WEI) for each sow. Sows were monitored daily for signs of estrus for 30 d post-weaning. Sows that did not exhibit estrus within this timeframe were culled.

### Chemical analysis

Grain and diet samples were collected. Samples of both corn and grain sorghum were collected at 3 timepoints only on the ground grain at the mill immediately after grinding. Diet samples were collected on every load through streamline sampling at discharge as feed trucks were loaded before delivery to the farm. Both grain and diet samples were stored at −20°C until they were homogenized, subsampled, and submitted for analysis. Grain and diet samples were sent for the determination of amino acids (AOAC 994.12), moisture (AOAC 930.15), crude protein (AOAC 990.03), ether extract (AOAC 2003.05), minerals (AOAC 985.01), and ash (AOAC 942.05; Midwest Laboratories, Omaha, NE; Tables 3 and 4).

**Table 3.** Chemical analysis of grains (as-fed basis).^a^

Item	Corn	Sorghum
**DM^b^, %**	83.8	85.9
**CP^c^, %**	6.5	9.6
**EE^d^, %**	3.9	2.7
**Ash, %**	0.87	1.25
**Ca, %**	<0.01	0.02
**Mg, %**	0.09	0.14
**P, %**	0.25	0.27
**K, %**	0.25	0.36
**Na, %**	<0.01	<0.01
**S, %**	0.08	0.09
**Cu, mg/kg**	1.1	2.4
**Fe, mg/kg**	12.7	28.1
**Mn, mg/kg**	3.8	15.0
**Zn, mg/kg**	23.1	19.0
**Amino acids, %**		
** Ala**	0.39	0.72
** Arg**	0.28	0.32
** Asp**	0.38	0.54
** Cys**	0.12	0.16
** Glu**	0.97	1.82
** Gly**	0.22	0.27
** His**	0.16	0.19
** Ile**	0.17	0.28
** Leu**	0.59	1.00
** Lys**	0.19	0.20
** Met**	0.13	0.14
** Phe**	0.23	0.39
** Pro**	0.45	0.72
** Ser**	0.26	0.39
** Thr**	0.17	0.24
** Trp**	0.05	0.11
** Tyr**	0.17	0.24
** Val**	0.24	0.35

a A samples of each grain source was homogenized, subsampled, and submitted for proximate analysis, mineral analysis and amino acid profile (Midwest Labs, Omaha, NE). Values represent the mean of 3 samples collected throughout the study.

b DM, dry matter.

c CP, crude protein.

d EE, ether extract.

**Table 4:** Chemical analysis of diets (as-fed basis).^a^

Item, %	Grain source		
	Corn	50:50 blend	Grain sorghum
**DM^b^, %**	86.7	87.1	87.6
**CP^c^, %**	18.1	18.4	19.7
**EE^d^, %**	3.2	4.3	3.4
**Ash, %**	3.4	4.8	4.7
**Ca, %**	1.08	0.97	0.99
**Mg, %**	0.16	0.16	0.18
**P, %**	0.52	0.48	0.50
**K, %**	0.86	0.85	0.84
**Na, %**	0.26	0.21	0.23
**S, %**	0.22	0.22	0.24
**Cu, mg/kg**	38.6	37.7	34.4
**Fe, mg/kg**	154	193	224
**Mn, mg/kg**	85.5	88.8	87.4
**Zn, mg/kg**	171	137	162

a A sample of each diet was homogenized, sub-sampled, and submitted for proximate analysis and mineral analysis (Midwest Labs, Omaha, NE). Values represent the mean of 7 samples collected throughout the study.

b DM, dry matter.

c CP, crude protein.

d EE, ether extract.

### Statistical analysis

Performance data were analyzed as a generalized block design using R software, version 4. 4. 1 (R Core Team, Vienna, Austria). Sow was considered the experimental unit. Dietary treatment and feeder treatment served as fixed effects with random effects of block (sow parity nested within farrowing group) and farrowing group. Dietary treatment and feeder treatment interactions were tested and were non-significant; therefore, this manuscript will only discuss the effects of dietary treatment. Pre-planned linear and quadratic contrast statements were used to evaluate increasing grain sorghum as a partial or complete replacement of corn in the diet. Pre-weaning removals and mortality were analyzed using a binomial distribution. Results were considered significant at *P* ≤ 0.05 and marginally significant at 0.05 < *P* ≤ 0.10.

## Results

### Ingredient and diet analysis

Subsequent chemical analysis of the grain sources confirmed that the analyzed values for AA, DM, crude protein, ether extract, and ash were similar to the values used in formulation (Table 3). Additionally, for the complete diets, results of chemical analysis indicated nutrient profiles were consistent with expected formulated values (Table 4). The target particle size for both grains was 500 μm with the resulting particle size for corn averaging 504 μm and sorghum averaging 442 μm throughout the study. However, all dietary particle sizes were within a 30 μm range (Table 2).

### Sow and litter performance

There were no differences in sow body weight, backfat, or caliper score at entry or weaning, or the change from entry to weaning between dietary treatments (Table 5). No differences were observed for sow average daily feed intake (ADFI) from farrowing to weaning. Additionally, there were no differences in sow wean-to-estrus interval as grain sorghum increased.

**Table 5.** Comparison of grain sorghum with corn in lactation diets on sow performance.^a^

Item	Grain source				*P* =	
	Corn	50:50 blend	Grain sorghum	SEM	Linear	Quadratic
**Count, *n***	190	189	189	—	—	—
**Parity**	2.5	2.5	2.5	0.51	0.692	0.920
**Lactation length, d**	19.3	19.2	19.3	0.84	0.682	0.423
**Sow BW^b^, kg**						
** Entry**	281.9	282.5	282.2	12.17	0.913	0.882
** Weaning**	243.8	246.4	245.0	12.91	0.657	0.399
** Change (entry to weaning)**	−38.4	−36.1	−37.3	1.53	0.494	0.201
**Sow backfat, mm**						
** Entry**	16.9	16.9	16.8	1.09	0.536	0.893
** Weaning**	13.6	13.9	13.6	0.64	0.948	0.306
** Change (entry to weaning)**	−3.4	−3.1	−3.2	0.55	0.576	0.335
**Sow caliper score, units**						
** Entry**	17.6	17.7	17.6	0.35	0.655	0.728
** Weaning**	16.1	16.4	16.4	0.41	0.261	0.576
** Change (entry to weaning)**	−1.4	−1.3	−1.3	0.17	0.316	0.722
**Sow ADFI^c^, kg**						
** Entry to weaning**	5.4	5.4	5.4	0.25	0.830	0.957
** Farrow to weaning**	6.6	6.7	6.7	0.27	0.850	0.760
**Wean to estrus interval, d**	5.6	5.5	5.7	0.14	0.364	0.182

a A total of 568 mixed-parity sows and litters (PIC 337 × 1050, Hendersonville, TN) were used from farrowing until weaning. Litters were cross-fostered across dietary treatment to equalize litter size up to 24-h post-farrowing.

b BW, body weight.

c ADFI, average daily feed intake.

For litter performance, there were no differences in total born or born alive (Table 6). The percentage of stillborn piglets decreased (linear *P* < 0.001; quadratic, *P* = 0.003) with increasing grain sorghum compared to corn. The percentage of mummies was greatest (quadratic, *P* = 0.001) in the 50:50 blend diet, with lower values in both the corn and grain sorghum diets. There were no differences in litter size on d 1 or at weaning. There were also no differences observed for litter weight, mean piglet weight, litter average daily gain (ADG), piglet ADG from d 1 to weaning, or litter feed efficiency.

**Table 6:** Comparison of grain sorghum with corn in lactation diets on litter performance.^a^

Item	Grain source				*P* =	
	Corn	50:50 blend	Grain sorghum	SEM	Linear	Quadratic
**Total born, *n***	17.1	16.6	16.8	0.46	0.497	0.321
**Total born alive, *n***	15.8	15.3	15.7	0.43	0.968	0.204
**Stillborn, %**	5.0	4.7	3.5	0.40	<0.001	0.003
**Mummy, %**	2.0	2.5	2.1	0.31	0.453	0.001
**Litter size, *n***						
** d 1**	14.9	14.9	14.8	0.36	0.769	0.978
** Weaning**	13.1	13.1	12.9	0.31	0.547	0.660
**Litter weight, kg**						
** d 1**	22.4	22.8	22.3	0.32	0.792	0.255
** Weaning**	71.0	70.2	68.9	3.99	0.174	0.831
**Litter feed efficiency^b^**	0.403	0.399	0.395	0.0099	0.503	0.974
**Mean piglet BW^c^, kg**						
** d 1**	1.51	1.54	1.51	0.045	0.903	0.223
** Weaning**	5.42	5.37	5.35	0.414	0.431	0.757
**Litter ADG^d^, kg**						
** d 1 to weaning**	2.65	2.61	2.55	0.095	0.204	0.910
**Piglet ADG, g**						
** d 1 to weaning**	202	198	197	0.01	0.338	0.864
**Mortality, d 1 to weaning, %**	7.6	6.4	7.3	0.52	0.606	0.080
**Removals, d 1 to weaning, %**	4.4	5.1	5.6	0.49	0.046	0.797
**Removals and mortality, d 1 to weaning, %**	12.0	11.5	12.8	0.75	0.362	0.202

a A total of 568 mixed-parity sows and litters (PIC 337 × 1050, Hendersonville, TN) were used from farrowing until weaning. Litters were cross-fostered across dietary treatment to equalize litter size up to 24-h post-farrowing.

b Litter feed efficiency = Total litter weight gain/total feed disappearance.

c BW, body weight.

d ADG, average daily gain.

There was a tendency for a decrease then increase (quadratic, *P* = 0.080) in preweaning mortality, with the lowest mortality in the 50:50 blend diet. Additionally, there was an increase (linear, *P* = 0.046) in preweaning removals as the grain sorghum increased in the diet. However, there was no difference in total preweaning removals and mortality between dietary treatments.

## Discussion

Historically, the use of grain sorghum in lactation diets has had variable results on sow performance (Louis et al. 1991; Johnston et al. 1998; Kim et al. 2002; Sotak-Peper et al. 2015). Both swine and grain sorghum genetics have changed over time, and previous research was not conducted under commercial sow production conditions, highlighting the need to re-evaluate the use of modern sorghum in lactation diets. Grain sorghum varieties used in previous research ranged from bronze to red in color and were predominantly low-tannin to tannin-free (Louis et al. 1991; Sotak-Peper et al. 2015), whereas the current study used a tannin-free sorghum variety. Sorghum is generally accepted as being tannin-free when values are at or below 4.0 mg of catechin equivalents per g of sample (U.S. Grains and Bioproducts Council 2026). Although grain sorghum color has historically been associated with tannin concentration, modern sorghum varieties often have low tannin levels regardless of kernel color, reducing the reliability of color as a predictor of feeding value (Boren and Waniska 1992). Since 2019, all U.S. grain sorghum varieties for feed use are tannin-free, indicating the need for updated research (U.S. Grains and Bioproducts Council 2026). Louis et al. (1991) evaluated either a corn-soybean meal or grain sorghum-soybean meal-based diet during gestation and lactation in a 2 × 2 factorial arrangement. Sows fed the grain sorghum-soybean meal diet during lactation had a 10% reduction in ADFI compared to sows fed the corn-soybean meal diet. This differs from the current study, which observed no differences in ADFI during lactation, regardless of grain sorghum level in the diet. The difference in ADFI observed in the present study compared to Louis et al. (1991) may be attributed to differences in feed processing and diet formulation methods. In the present study, similar dietary particle size across treatments and standardized ileal digestibility of amino acids were used in diet formulation, whereas Louis et al. (1991) used diets with particle sizes ranging from 975 to 1,191 μm and total amino acids for diet formulation.

Johnston et al. (1998) evaluated grain source (corn vs. grain sorghum) and conditioner procedure (mash vs. standard steam conditioner pellets vs. expanded pellets) and observed a tendency for sows fed the grain sorghum-based diet to have a greater ADFI during lactation compared to sows fed the corn-based diet, regardless of conditioner practice. However, sows in their study were fed sorghum-based gestation diets, while in the current study, sows were provided a wheat-based diet during gestation. To prevent any bias towards a specific grain, sows were offered a wheat-based gestation diet in the present study to eliminate any potential concern of an interaction between gestation grain source and lactation grain source. Sotak-Peper et al. (2015) evaluated lactation diets containing either corn or grain sorghum with or without 20% grain sorghum distillers dried grain with solubles (DDGS). There was no difference in sow ADFI during lactation regardless of grain source, independent of DDGS level. While previous studies with grain sorghum in lactation diets have had variable effects on sow ADFI, the grain sorghum variety used in the present study can be implemented without negatively affecting lactation feed intake.

Given that lactation feed intake influences the mobilization of maternal body reserves, sow bodyweight and backfat change from entry to weaning was also evaluated. No differences were observed in sow BW change from entry to weaning in the current study, which agrees with Louis et al. (1991) and Johnston et al. (1998). However, in the study by Sotak-Peper et al. (2015), when assuming similar energy values for sorghum and corn, sows fed grain sorghum-based diets lost less weight during lactation compared to those fed corn-based diets. Because the corn and grain sorghum in diets used in the current study were assumed to be identical and there was no change in ADFI, this likely explains the lack of differences observed in sow BW change. Additionally, there were no differences observed in backfat (BF) change, which is in agreement with both Johnston et al. (1998) and Sotak-Peper er al. (2015). Furthermore, no differences were observed in WEI, which agrees with findings of Louis et al. (1991) and Johnston et al. (1998).

For farrowing measurements, no differences were observed in total born alive. However, there was a quadratic increase and then decrease in the percentage of mummified pigs as grain sorghum increased. This differs from the study conducted by Louis et al. (1991), which observed no differences in the number of mummies regardless of grain source. Because diets in the present study were introduced on d 112 of gestation, the difference in mummies is likely not related to dietary treatment. Additionally, there was a quadratic decrease observed in the percentage of stillborn pigs as grain sorghum increased. This is also in contrast with Louis et al. (1991) in which no differences were observed in the number of stillborns regardless of grain source. Although the reductions in stillborns are at a magnitude of industry relevance, further research is needed to distinguish if this response is related to dietary factors included in sorghum or potential differences in farrowing management, farrowing duration, or piglet birth order, as well as to replicate it in other commercial settings.

The lack of difference in litter weaning weight, piglet weaning weight, and litter or piglet gain for sows fed corn- or grain sorghum-based diets is similar to results of Johnston et al. (1998) and Sotak-Peper et al. (2015). Conversely, Louis et al. (1991) observed piglets weaned from sows fed the grain sorghum-soybean meal-based diet gained less weight and were lighter at weaning. Their results may be explained by the decreased ADFI and N digestibility for sows fed grain sorghum-based diets, combined with the greater particle size of their complete diet containing grain sorghum, which would have reduced protein and energy digestibility (Owsley et al. 1981). These differences between the corn and grain sorghum diets could help explain the variation in responses seen compared to the present study where diets had similar particle size. Corn and grain sorghum were ground to 500 μm in the current study to prevent any whole grain sorghum berries from entering the complete feed. In addition to piglet growth, there were no differences observed in litter size at weaning or total removals and mortality, which agrees with Louis et al. (1991), Johnston et al. (1998) and Sotak-Peper et al. (2015).

## Conclusion

In conclusion, there was minimal impact on sow and litter performance as grain sorghum replaced corn as the cereal grain in the lactation diet. Based on the current data, tannin-free grain sorghum grown and harvested in the United States can be used to partially or fully replace corn in lactation diets when economically justified.

## List of abbreviations

AA: amino acids
ADG: average daily gain
ADFI: average daily feed intake
BF: backfat
BW: body weight
DM: dry matter
FCR: feed conversion ratio
ME: metabolizable energy
NE: net energy
RFID: radio frequency identification
SID: standardized ileal digestible
STTD: standardized total tract digestible
WEI: wean-to-estrus interval
